# 

**Published:** 1908-07

**Authors:** 


### BOOKS RECEIVED.

State Board Questions and Answers. By R. Max Goepp, M.D., professor of clinical medicine at the Philadelphia Polyclinic. Octavo, pp. 684. Philadelphia and London: W. B. Saunders Co. (Cloth, $4.00.)

Adenomyoma of the Uterus. By Thomas Stephen Cullen, M.D. associate professor of gynecology in the Johns Hopkins University. Royal 8vo, pp. 270. Illustrated. Philadelphia and London: W. B. Saunders Co. (Cloth, $5.00.)

The Principles and Practice of Hydrotherapy. By Simon Baruch, M.D., professor of hydrotherapy in Columbia University (College of Physicians and Surgeons) New York. Third edition. Octavo, pp. 544. Illustrated. New York: William Wood & Co. (Cloth, $4.00.)

Mellin's Food Method of Percentage Feeding. 12 mo., pp. 183. Illustrated. Boston: Mellin's Food Co. 1908.

Subcutaneous Hydrocarbon Protheses. By F. Strange Kolle, M.D., Author of the "Recent Roentgen Discovery," etc. 12 mo, pp. 153. New York: The Grafton Press. (Cloth, $2.50.)

Insomnia and Nerve Strain. By Henry S. Upson, M.D., professor of diseases of the nervous system in the Western Reserve University, Cleveland, O. 12 mo, pp. 142. Illustrated. New York and London: G. P. Putnam's Sons. 1908.

Modern Medicine. Its Theory and Practice. Edited by William Osler, M.D., Regius Professor of Medicine in Oxford University, Eng. Assisted by Thomas McCrea, M.D., Associate Professor of Medicine and Clinical Therapeutics in Johns Hopkins University, Baltimore. In seven octavo volumes of about 900 pages each, illustrated. Volume IV. Lea & Febiger: Philadelphia and New York, 1908. (Cloth, $6.00, leather, $7.00, half morocco, $7.50 net.)

Progressive Medicine, Vol. X, June, 1908. A Quarterly Digest of Advances, Discoveries and Improvements in the Medical and Surgical Sciences. Edited by Hobart Amory Hare, M.D., Professor of Therapeutics and Materia Medica in the Jefferson Medical College of Philadelphia. Octavo, 352 pages. Lea Brothers & Co: Philadelphia and New York. (Per annum in four cloth-bound volumes, $9.00; in paper binding, $6.00; carriage paid to any address.)

---

### LITERARY NOTES.

### INDEX TO VOL. LXIII.

### AUGUST, 1907—JULY, 1908.

Page

ABDOMINAL pregnancy ........ 30
Abscesses, multiple post-typhoid 332
Academy, our ..................... 63
Actinomyces infection ............. 133
Adrenalin during ether anesthesia.. 95
Albany sanitary bureau ........... 229
Alcohol, scientific use of .......... 20
Anesthesia, danger signals in ...... 205
Aorta, aneurism of ............... 163
Apollinaris and champagne ........ 480
Appendicitis, operation for under difficulties ........................ 417
Aseptic operative technic .......... 137

BACTERIAL vaccines ........... 692
Bainbridge, William Seaman, transmissibility and curability of cancer .......................... 19
Banknotes, malignant scarlet fever from ........................... 478
Bender, Ida C., and the Second International Congress of School Hygiene ........................ 168
Bergey, D. H., opsonins and vaccines as applied to surgical therapy 85
Biliary affections, treatment of ..... 663
Bock, Franklin W., physical defects in children ...................... 67

**Books and Authors:**

Aitkens: Training school methods .......................... 367
Anders: Physical diagnosis ... 181
Anders: Practice of medicine.. 556
Babcock: Diseases of the lungs. 118
Barnhill and Wales: Otology... 555
Baumann: Gonorrhea ......... 721
Bishop: Heart disease and blood pressure ..................... 491
Blair: Materia medica ........ 306
Boas: Diseases of stomach .... 425
Brickner: Surgical suggestions. 495
Brinkerhoff: Diseases of rectum 431
Brown: Medical diagnosis ..... 122
Bryant and Buck: Surgery, vol. 3, 246; vol. 4 ............... 724
Budin: The nursling .......... 121
Bulkley: Diseases of skin ...... 121
Crossen: Diseases of women ... 427
DaCosta: Surgery ............ 305

Page

Delafield and Prudden: Textbook of pathology ........... 360
Dorland: Medical dictionary .. 307
Drew: Invertebrate zoology ... 369
Edwards: Practice of medicine. 179
Egbert: Hygiene .............. 490
Eisendrath: Clinical anatomy .. 429
Eisendrath: Surgical diagnosis. 301
Ferguson: Diseases of nose and throat ....................... 183
Ferguson: Modern operations for hernia .................. 53
Fischer: Diseases of infancy.... 358
Foote: Minor surgery ......... 618
Fowler: Operating room and patient ...................... 431
French: Practice of medicine... 361
French: Syphilis in the army .. 554
Gilliam: Gynecology .......... 550
Gilliam: Rose Croix .......... 57
Gleason: Nose, throat and ear.. 180
Greene and Brooks: Genitourinary organs and kidney ....... 492
Greene: Kirke's physiology .... 362
Gouley: Genitourinary diseases. 494
Griffith: Care of baby ........ 306
Hallenberg: Pharmacopeia and national formulary .......... 554
Hare: Practical therapeutics ... 362
Hare: Practical diagnosis ..... 428
Hare: Practice of medicine.... 120
Hare: Progressive Medicine, June, 1907, 181; September, 1907, 243; December, 1907, 486; March, 1908 ............ 618
Hardaway and Grindon: Cutaneous therapeutics ............ 487
Head: Practical Medicine Yearbooks, 1906, vol. X, skin and venereal diseases, 59; 1907, vols. 1, 2, 3.................. 357
Hecker: Diseases of children .. 307
Heisler: Textbook of embryology .................. 367
Howell: Textbook of physiology ...................... 366
Hoxie: Medicine for nurses ... 553
Hutchison: Index of treatment 724
Jackson: Diseases of the eye... 303
Jennings: Hygiene of pregnancy 59
Jewett: Essentials of obstetrics 58
Jewett: Practice of obstetrics... 368

Page

Keen: Surgery, vol. 2, 244; vol. 3 .......... 617
Kelly: Gynecology and abdominal surgery, vol. 1 .......... 359
Kerr: Diseases of children .......... 360
Keyes: Syphilis .......... 722
King: Manual of obstetrics .......... 123
Kyle: Diseases of nose and throat .......... 556
Lea: Practitioners' visiting list, 1908 .......... 302
Lippincott: International Clinics, 17th series, vol. 2, 182; vol. 3, 246; vol. 4, 494; 18th series, vol. 1 .......... 675
Little: Nephritis .......... 369
Luckett: Paraffin in surgery.......... 119
McCaw: Diseases of children .......... 122
McCombs: Diseases of children 549
McFarland: Pathogenic bacteria 120
May: Diseases of eye .......... 431
Meyer: Bier's hyperemic treatment .......... 725
Miller: Cosmetic surgery .......... 554
Mills: Tumors of the cerebrum. 57
Modern Clinical Medicine: Nervous system .......... 488
Moore: Medicine in British Isles 676
v. Neusser: Disorders of respiration and circulation .......... 365
Nothnagel: Practice of Medicine, diseases of heart, 488; second edition, vol. 1 .......... 430
Osler: Modern medicine, vol. 1, 176; vol. 2, 364; vol. 3.......... 551
Ott: Textbook of physiology .......... 363
Overall: Diseases of prostate gland .......... 56
Park: Modern surgery .......... 237
Paul: Materia medica for nurses 303
Piersol: Human anatomy .......... 357
Pottinger: Pulmonary tuberculosis .......... 675
Pusey: Dermatology .......... 178
Pyle: Personal hygiene .......... 300
Ravogli: Syphilis .......... 720
Register: Fever nursing .......... 430
Reissig and Jelliffe: Standard family phyiscian .......... 244
Robson: The pancreas .......... 493
Sajous: Internal secretions, vol. 2 .......... 366
Savage: Insanity .......... 550
Savill: Neurasthenia .......... 368
Schafer: Histology .......... 304
Schleif: Materia medica and therapeutics .......... 369
Scudder: Treatment of fractures .......... 363
Scott: Sexual instinct .......... 549
Simon: Clinical diagnosis .......... 365
Simon: Physiological chemistry 490

Page

Sobotta: Atlas and textbook of anatomy, vol. 3.......... 548
Stelwagon: Diseases of skin .......... 555
Stevens: Practice of medicine .......... 491
Stimson: Fractures and dislocations .......... 429
Strong: Electrotherapeutics .......... 553
Sutherland: Blood stains .......... 367
Talmey: Woman .......... 489
Treves: Surgical anatomy .......... 550
Underwood: Aids to dental surgery .......... 123
Wainwright: Medical knowledge of Shakespere .......... 548
Wallian: Rhythmotherapy .......... 54
Webster: Diseases of women .......... 56
Wells: Chemical pathology .......... 245
Whiting: Aids to medical diagnosis .......... 123
Wilson: Obstetric nursing .......... 554
Williams: Textbook of obstetrics .......... 426
Witthaus: Chemistry and toxicology .......... 183
Witthaus and Becker: Medical jurisprudence, vol. 2 .......... 300
Wood: Diseases of eye .......... 492
Wood: Medical Record visiting list, 1908 .......... 302

**Reports:**

Conferences of state health officers with United States public health and marine hospital service, 1905-06 .......... 59
Commissioner of Education, vols. 1 and 2. 1905.......... 306
Johns Hopkins Hospital, vols. 13 and 14 .......... 182
State Department of Health, N. Y., 1905.......... 307
Surgeon-general public health and marine hospital service, 1906 .......... 58

**Transactions:**

American Dermatological Association, 1906 .......... 305
American Surgical Association, 1907 .......... 676
American Urological Association, 1907 .......... 726
Books Received 59, 124, 183, 247, 308, ........369, 432, 495, 557, 619, 676, 725
Boswell, Charles O., dibotrocephalus latus infection .......... 511
Bonifield, Charles L., retrodeviations of uterus .......... 584

Page

Bovée, J. Wesley, position of surgery in treatment of peritonitis .. 377
Bronchopneumonia, colloidal silver in 96
Bull, W. Sheldon, our backyard dairy 577
Bulletin Buffalo department of health 541
Buttermilk, homemade ............ 217

CALCULI, vesical, how often overlooked? ................. 1
Calox, oxygen tooth powder ..... 345
Cancer, of breast, danger of pregnancy following operations for .............. 314
transmissibility and curability of .................... 19
treatment of ............ 311
Cannaday, John E., abdominal pregnancy ........ 30
discussion on perineal tears 77
aseptic operative technic.. 137
strangulated inguinal hernia in infants 204
tubes and ovaries, treatment of .......... 600
Cesarean section, vaginal .......... 644
Chaillé Jubilee .................... 703
Champagne, Moet & Chandon White Seal ........................... 345
Cheesman, William S., danger of pregnancy after operations for cancer of breast ................. 314
Children, physical defects in........ 67
Cholelithiasis, therapeusis of....... 409
Chorea, treatment of .............. 637
Class reunions .................... 713
Colegrove, B. H., extracts from a doctor's ledger .................. 450
Collargolum ....................... 289

**College and Hospital Notes:**

Buffalo General Hospital ...... 485
Buffalo General Hospital, surgical clinics .................. 174
Children's Hospital, Rockaway Beach ...................... 175
College of Medicine, Syracuse University ..............118, 174
Columbus Hospital ............ 719
German Hospital, Buffalo ...... 116
German Deaconess's Hospital, Buffalo .................485, 616
Hahnemann Hospital. Buffalo 356, 485
Hospital ship "Buffalo" ....... 674
Hospital for Deformities and Joint Diseases, New York.... 356
Hospital Internes, appointments of ......................... 720
Kenerson Private Hospital ..... 719
Lockport City Hospital 485, 616, 674
Louisville Medical College and Hospital College of Medicine, merger between ..........237, 425
Marine Hospital. Buffalo 117, 356 617
Medina Hospital ............... 486
New York City Hospital ....... 485
New York Post-Graduate Hospital ....................... 237
New York Skin and Cancer Hospital ................237, 547
Polish Hospital, Buffalo...... 117
Tulane Alumni ............... 617
Tulane University ............ 616
University of Buffalo, Medical Department .........117, 486, 615
University of Buffalo commencement ........................ 674
University of Pennsylvania Medical School ............. 424
Yale Medical School .......... 547

Conviction under food and drug law 540

**Correspondence:**

Alcoholic problem—T. D. Crothers ......................... 534
Experimental medicine, committee on—Bryant-Curtis ....... 474
Sanitary milk problem—Walter Wyman ..................... 164
Smithsonian Institution, Hodgkins fund prize—Charles D. Walcott .................... 474
Substituting in Chicago—Fairchild Bros. & Foster ........ 225

DAIRY, our backyard ........... 577
Daly, James R. L., feeding in tuberculosis .................... 440
Degeneration, mental stigmata of... 8
Dibotrocephalus latus infection ..... 511
Digestive apparatus, testing functions of ......................... 473
Diploma, sentimental value of ..... 677
Doctor's ledger, extracts from...... 450
Dogs in French army medical corps. 345
Dowd, J. Henry, acinitis prostatica.. 267
Draper, Andrew S., anniversary dinner to ......................... 714

ECHINOCOCCUS cyst of the liver 383
Erie County Hospital, reforms suggested ...................... 46
Everyday lessons from everyday cases ......................... 459

Page

Ewart, William, treatment of pneumonia ... 279
Eye strain, epilepsy caused by ... 559

FERMENLACTYL, old age and .. 603
Fitch, W. E., treatment of uterine hemorrhage ... 497
Fly, danger of house ... 714
Food and drug laboratory ... 668
Forceps, obstetric ... 274
Foxwell, Arthur, some new sedatives 78
Fraternity night ... 712
Frye, Maud J., woman in medicine. 233

GARDNER, James A., how often are vesical calculi overlooked?. 1
Germ scare, a new ... 229
Goitre, serum treatment of exophthalmic ... 287
Gonococcus, puerperal infection from 211
Gonorrhea, internal medication in.. 412
treatment of with arhovin ... 410
Gould, George M., epilepsy caused by eye strain ... 559
Greater University ... 608
almshouse farm offered to ... 668
dinner for ... 479

HARRISON, President, a story of 230
Hayd, H. E., echinococcus cyst of the liver ... 383
Hay fever ... 149
Health, department of and President Roosevelt ... 418
Hernia, strangulated inguinal in infants ... 204
Honor, modern standard business 16
Hospital interne ... 632
Hyperchlorhydria, relation of to motor function of stomach ... 316

ILLUSTRATIONS:
Gardner, vesical calculi ... 2
LeBreton, lateral curvature of the spine:
Fig. 1, adolescent type ... 199
Fig. 2, curvature from unilateral hypertrophy ... 200
Fig. 3, adolescent type of mild grade ... 200
Fig. 4, curvature due to sciatica ... 201
Fig. 5, kyphosis and scoliosis ... 201
Fig. 6, deformity due to empyema ... 202
Fig. 7, curvature due to paralysis ... 203

Page

Bull, our backyard dairy:
Fig. 1, young Saanen doe ... 579
Fig. 2, goat barn ... 581
MacLeod, coagulation tube ... 629
Roosa, D. B. St. John, portrait 611

Influenza of nose, throat and larynz 413
renal complication of ... 413
Insane, the partially ... 157
Intravenous injection of medicaments 601

Items:

Battle & Co., Dislocations ... 61, 248, 372, 620
Blackwell's Island ... 186
Breitenbach Co., M. J.—Physician's Daily Reminder ... 372
Civil service commission examinations, N. Y. state ... 185, 248, 433, 558, 620
Civil service commission examinations, U. S. ... 185, 372, 678
Death sentence, instrument used 62
Denver Chemical Manufacturing Co.,—note and card case ... 371
Drevet Manufacturing Co.,—Meatox ... 185
Panama canal examinations for physicians ... 434
Public Health and Marine Hospital Service examinations ... 372
Substitution knocked out in New York ... 62
Switzerland peasant not of good physique ... 62

JENKS, Jeremiah W., modern standard business honor ... 16
Jewett, Charles Sherman, our Academy ... 63
Jones, Allen A., medicine a science. 187
hyperchlorhydria and motor function of stomach. 316
William B., treatment of cancer ... 311
Judson, Adoniram B., recumbency in treatment of infantine paralysis 75

KOCH, Robert, entertainment for. 541

LACTIC ferments, therapy of ... 408
Lanoline as an excipient ... 157

Leading Articles:

Appetite, digestion and health .. 665
American Medical Association at Atlantic City ... 43

Page
American Association of Obstetricians and Gynecologists. 165
Army medical bill .....292, 415, 536
Antivivisection bill ............ 476
Carnegie honor for a physician. 540
Council on medical education, conference of ............... 606
Daniel Bennett Saint John Roosa ....................... 535
Dr. Kocher's visit to Albany... 45
Doctor in politics ............. 338
Folia Therapeutica ............. 110
Greater University ............ 337
Hygienic kitchens ............ 108
Mr. Bok's attack .............. 539
Modern cystoscopy ............ 414
Pilcher, James E., president, etc 109
Pirrie, Alexander Mactier ..... 342
Responsibility of health on high seas ........................ 538
Ryan, Frank G., president of Parke, Davis & Co.......... 47
Squibb's Materia Medica ...... 341
State sanitary conference ...... 226
University of Buffalo, 1846-1898 710
Vivisection bill ............... 416
Wellcome's Photographic Exposure ....................... 340

LeBreton, Prescott, lateral curvature of the spine .................... 199
Legal pharmacy .................. 156
Leidy, Joseph, statue of ........... 295
Lewis, F. Park, ophthalmia neonatorum ......................... 321
Light as a health maker ........... 213
Lipoma arborescens of knee joint... 444

**Literary Notes:**

Albany Medical Annals ........ 433
American Journal of Obstetrics. 726
American Medicine ........... 496
Anglo-American Medical Association, report of ............ 371
Archives of Diagnosis ......... 496
Burrell's Surgery, P. Blakiston's Son & Co................... 124
Boston Medical and Surgical Journal ...................... 678
Columbus Medical Journal .... 433
Davis's Biography, by Danforth —Cleveland Press of Chicago 124
Education building, state....... 726
Fort Wayne Medical Journal-Magazine .................... 433
Indiana State Medical Association Journal ................ 433
Interstate Medical Journal ..... 61
Jacobson's Operations of Surgery—P. Blakiston's Son & Co 371
Lecture by Thomas Darlington. 248
National Hospital Record ..... 433
Our Own Folks, D. Appleton & Co ........................ 61
Parke, Davis & Co.,—brochure of pathologic laboratories ... 185
Physician's visiting list—J. H. Chambers & Co ............ 310
Saint Louis Medical Review ... 61
Saunders, W. B. Co.—Illustrated catalogue .................... 371
Saunders, W. B. Co.—Announcement ................ 677
School Hygiene ............... 726
Texas Medical News .......... 371
The Hospital ................. 247

Little, F. H., multiple post-typhoid abscesses ....................... 332
Liver, salicylic acid in pathologic conditions of .................. 95

MacDONALD, mental stigmata of degeneration ................ 8
MacLeod, James A., varicose veins. 621
Norman K., bacterial vaccines ................ 692
McMurtry, L. S., address at Chaillé Jubilee ........................ 703
McPhedran, early diagnosis of cancer of stomach ................. 506
Manton, W. P., weight relations of placenta and child .............. 562
Meatox .....................284, 602
Medical examination fee restored .. 111
experts, extra compensation for ..................... 219
inspection of schools, lack of in Buffalo ........... 344
Medicine a science ............... 187
Meisenbach, R. O., lipoma arborescens of knee joint ............. 444
Menorrhagia and metrorrhagia .... 650
Military Surgeons at Jamestown ... 294

**Miscellany:**

Alaska-Yukon-Pacific Exposition ........................ 558
Army medical corps examinations ....................... 496
Bowery mission ............... 558
Good Housekeeping ........... 620
Seaman prize ................. 620

Monell, S. H., present status of physiologic therapy ............. 435
Morrow, P. A., prophylaxis of social diseases ...................... 138
Mosquitos, campaign against....... 714

NARCOLEPSY, case of ......... 91
Navy hospital ships commanded by medical officers ............ 418
Nervous diseases, radiant light bath in 336
Neu, M., suprarenin in obstetrics.... 455
Nurses, trained, in navy ........... 608

Page

OBITUARY:
Alling, Charles P. . . . . . . . . . . . . . 672
Ashley, Harmon J. . . . . . . . . . . . . 717
Barton, Theodore Woodward . . 348
Battle, Cullen Andrews . . . . . . . . 613
Bevier, Matthew . . . . . . . . . . . . . 544
Bowen, Clara E. . . . . . . . . . . . . . . 421
Briggs, George H. . . . . . . . . . . . . . 422
Broadbent, Sir William Henry. . 50
Carroll, James . . . . . . . . . . . . . . . 172
Cartledge, Abiah Morgan . . . . . . 671
Casey, James W. . . . . . . . . . . . . . . 348
Chace, William Holden . . . . . . . . 544
Clapp, Henry D. . . . . . . . . . . . . . . 613
Clark, Gaylord Parsons . . . . . . . . 172
Crumb, Dewitt Clinton . . . . . . . . 672
Culbertson, James C. . . . . . . . . . 717
Davie, Charles H. . . . . . . . . . . . . 718
Dee, Robert Hill . . . . . . . . . . . . . . 544
Doherty, Edward P. . . . . . . . . . . . 422
Douglas, Richard . . . . . . . . . . . . . 543
Fassett, Bryant Sloat . . . . . . . . . 612
Hyde, Joel Wilbur . . . . . . . . . . . . 232
Houston, Samuel . . . . . . . . . . . . . 348
Laidley, L. H. . . . . . . . . . . . . . . . . 482
Lawrence, Arthur John. . . . . . . . 613
Loomis, Henry Patterson . . . . . . 349
McElhany, M. J. . . . . . . . . . . . . . . 422
Mann, Rev. Arthur S. . . . . . . . . . . 112
Markoe, Francis Hartman . . . . . 172
Miller, Frederick W. . . . . . . . . . . 717
Morris, Richard . . . . . . . . . . . . . . 349
Mosshammer, Jesse C. . . . . . . . 717
Moyer, Frank H. . . . . . . . . . . . . . . 172
Oliver, James Albert . . . . . . . . . . 349
Pickard, Peter . . . . . . . . . . . . . . . . 232
Powell, Seneca D. . . . . . . . . . . . . . 114
Proctor, George Monroe . . . . . . . 482
Putnam, J. W. (Lyons, N. Y.) . 481
Ring, Charles A. . . . . . . . . . . . . . . 543
Robinson, Albert B. . . . . . . . . . . . 612
Schugens, M. Elizabeth . . . . . . . . 419
Memorial . . . . . . . . . . . . . . . 232
Senn, Nicholas . . . . . . . . . . . . . . . 420
Shrady, George Frederick . . . . . 347
Sharp, H. P. . . . . . . . . . . . . . . . . . . 482
Stoddart. Enoch V. . . . . . . . . . . . . 718
Tanner, Herbert N. . . . . . . . . . . . . 482
Thornbury, Frank J. . . . . . . . . . . . 613
Trowbridge, Grosvenor R. . . . . . 670
Turrill, General Henry S. . . . . . . 49
Tweedy, Edward H. . . . . . . . . . . . 672
Waldron, George . . . . . . . . . . . . . 172
Whipple. Electa B. . . . . . . . . . . . . 171
Memorial . . . . . . . . . . . . . . . 482
White, Ahira R. . . . . . . . . . . . . . . . 422
Wise, Peter M. . . . . . . . . . . . . . . . . 171
Wood, William H. S. . . . . . . . . . . 348
Wynn, Webster W. . . . . . . . . . . . . 544

Obstetrics, neglect of scientific . . . . . 125
"Old home week" . . . . . . . . . . . . . . . . 111
Opsonins and vaccines as applied to surgical therapy . . . . . . . . . . . . . . . . 85

Page
Optometry bill . . . . . . . . . . . . . . . . . . 542
signed . . . . . . . . . . . . 669
veto of . . . . . . . . . . . . 111
Ophthalmia neonatorum, a pathologic anachronism . . . . . . . . . . . . . . . 321

PANCREATITIS, acute . . . . . . . . 689
Paralysis, recumbency in treatment of infantile . . . . . . . . . . . . . . . . 75
Parmenter, F. J., actinomyces infection . . . . . . . . . . . . . . . . . . . . . . . . . . . 133
Perineal tears . . . . . . . . . . . . . . . . . . . 77
Peritonitis, opium treatment of. . . . . 373
position of surgery in. . . 377

**Personal:**

Brush, Edward N.; Greene, Walter D.; Grove, B. H.; Carroll, Jane W.; Millard, O.; Tate, Magnus A. . . . . . . . . . . . . 113
Curtis, Frederic C.; Frederick, Carlton C . . . . . . . . . . . . . . . . . . 230
Cathcart, Robert Spann; Bonifield, Charles L. . . . . . . . . . . . . . 232
Degries, J. Carlisle; Carroll, Jane W.; Lewis, Bransford; Millican, Kenneth W.; Wheeler, David E. . . . . . . . . . . . . . . . . 346
Dunham, S. A.; MacLeod, James A.; Cowper, H. W.; Leo-Wolf, Carl G.; Gaertner, William; Burke, Joseph; Murphy, John B. . . . . . . . . . . . . . 669
Gibson. James A.; Leo-Wolf, C. G.; Whitford, William; Gaertner, William; Pohl, Esther; Clark, Edward; Grosvenor, J. W.; Carroll, Jane W. . . . . . . . . . . . . . . . . . . . . . . . . . 231
Gorgas, William C.; Stockton, Charles G.; Hubbell, Alvin A.; Wende, Grover W.; Townsend, Wisner R.; Benedict, A. L.; Chase, Walter B.; Manton. Walter P. . . . . . . . . . . . 716
Grant, John H.; Pryor, John H.; Bagley, Thomas; Greene, Dewitt C. . . . . . . . . . . . . . . . . . . . 717
Hitzel, Gustave A.; Weidman, John A.; Cabot, Richard C. . . 480
Long, Eli H.; Williams, Herbert U.; Howell, Stephen Y.; Billings, William H.; Blanchard, Robert B.; Aldrich, John O.; Ely, C. F.; Hill, Herbert M. . 610
Mann, Dr. and Mrs. M. D.; Krauss, William C.; McMurtry, L. S.; Mathews. Dr. and Mrs. Joseph M.; Himmelsbach, G. A.; Vander Veer, A.; Walker, Edwin . . . . . . . . . . . . . 49
McMurtry, L. S.; Cannaday, John E. . . . . . . . . . . . . . . . . . . . . . 609

Page

Means, W. J.; Hengerer, A. W. 114
Meyer, A. W.; Richard, A. M.; Bull, Alexander T.; Lewis, F. Park ........................ 670
Mooney, James J.; Andrews, Herman D.; McMurtry, Lewis S.; Crego, Floyd S.; Strong, Thomas D. ................... 296
Putnam, James W.; Lewis, F. Parke; Krauss, William C.; Grant, John H.; Taneyhill, G. Lane; Gaertner, William; Drake, Francis A.; Longyear, Howard W.................. 170
Seaman, Louis L.; Andrews, Champe S.; Gipple, Benjamin A.; Morris, Robert T.; Ely, William S.; Loop, Ross G.; Lewis, F. Park ............. 481
Trick, Harry R.; Smith, Lawrence H ..................... 542
Runyan, Joseph P.; Ross, James F. W.; Grove, Benjamin H.. 171
Smith, Regent T. Guilford .... 169
Wilcox, Dewitt G.; Potter, William Warren; Koch, Robert; Wiley, Harvey W........... 612
Wilson, Nelson W.; Parmenter, William L. and F. J.; Lewis, F. Park .................... 295
Zinke, E. Gustav; Stokes, Charles F.; Keyes, E. L. Jr.; Burrell, Herbert L.; Bingham, H. H. ...................... 419

Pharmacists and physicians......... 286
Phipps Psychiatric Clinic .......... 713
Physiologic therapy, present status of 435
Pitman, Sir Henry Alfred, oldest physician in United Kingdom .... 230
Placenta and newborn child........ 562
Pneumonia, treatment of........... 279
Porter, Eugene H., sanitary work... 249
Practice act, Illinois amends ....... 344
Pregnancy, abdominal ............. 30
Prescribing vs. dispensing ......... 155

**Progress in Medical Science:**

Medico-legal, by E. S. McKee.. 218
Therapeutics, by E. S. McKee.. 23

Proprietary remedies, nomenclature of ............................. 164
Prostatica acinitis ................ 267
Puerperal infection, collargolum in. 410

RECTUM, villous papilloma of... 29
Red Cross, American National, used commercially .............. 290
Regents' appointments .........343, 715
Reed, Charles A. L., address by.... 715
Charles A. L., everyday lessons from everyday cases.. 459
Reid, Ambassador, address of...... 416

Page

Report of Champe S. Andrews .... 344
Ricketts, Benjamin Merrill, villous papilloma of the rectum ......... 29
Rogers, Bertram M. H., case of narcolepsy ........................ 91
Roosevelt, President, and the International Dermatological Congress 167

SAGE, Russell, Institute of Pathology ....................... 295
Sanitary work .................... 249
Savary, dangers of arising immediately upon awakening ........... 480
Sedatives, new .................... 78
Scarlet fever, treatment of ........ 387
Sciatica, therapy of ............... 23
Shurly, E. L., hospital interne ..... 632
Social diseases, prophylaxis of ... 138

**Society Meetings:**

Academy of Medicine, Elmira.. 424
Academy of Medicine, Rochester ...........424, 484, 545, 615, 673
Academy of Medicine, Syracuse 424
American Association of Obstetricians and Gynecologists..51, 114
American Medical Association.. 614
American Gastroenterological Society ..................... 547
American Hospital Association 173
American Medico-Psychological Association ................. 544
American Proctologic Society.. 547
American Public Health Association .................... 116
American Therapeutic Society . 614
Association of American Medical Colleges ................ 484
Association of Military Surgeons ...................... 296
Buffalo Academy of Medicine.. 235 299, 350, 423, 483, 545, 614, 673, 718
Buffalo General Hospital Alumni Association .............. 615
Dunkirk and Fredonia Medical Society ..................... 351
Fifth Pan-American Medical Congress ................422, 673
Homeopathic Medical Society State of New York ......... 485
International Congress of Tuberculosis .............236, 352, 673
International Dermatological Congress .................. 116
Lake Keuka Medical and Surgical Association .....53, 116, 718
Medical Association of Central New York ................. 173
Medical Meetings at Chicago .. 614
Medical Society County of Chautauqua ................. 351
Medical Society County of Erie ......................234, 613, 718

Page

Medical Society County of Genesee .................... 235
Medical Society County of Montgomery ................ 349
Medical Society County of Ontario ........................ 235
Medical Society County of Steuben .................... 236
Medical Society County of Wyoming ........................ 235
Medical Society County of New York ........................ 546
Medical Society State of New York ...............236, 423, 483
Medical Society State of New York, Sixth District Branch. 173
Medical Society State of New York, Eighth District Branch ...................... 53, 115, 234
Medical Union ................. 351
Mississippi Valley Medical Association ................116, 614
National Fraternal Congress... 115
New York and New England Association of Railway Surgeons ........................ 236
New York Medico-Legal Society 485
N. Y. State Conference of Health Officers ...........52, 173
Ohio Association of Medical Teachers ................... 423
Oregon State Medical Association ........................ 719
Sixteenth International Medical Congress .................. 354
Southern Surgical and Gynecological Association .173, 299, 423
Western Surgical and Gynecological Association ........ 424

**Society Proceedings:**

Medical Association of Central New York .................. 516
Medical Society County of Erie .....................282, 471, 658
Roswell Park Medical Club.... 528
Spine, lateral curvature of ........ 199
Stamm, vaginal Cesarean section .. 644
Sterility due to conical cervix and anteflexion ...................... 650
Stockton, Charles G., acute pancreatitis ............... 679
Charles G., opium treatment of peritonitis .... 373
Stomach, early diagnosis in cancer of ........................... 506
Substitution as a penal offense .... 220
"Sundown doctors" .............. 112
Suprarenin in obstetrics ........... 455
Syphilis, professional secrecy in .... 153
and massage ............. 210

THEODORE, Duke Carl of Bavaria, only royal doctor in Europe ......................... 46

Page

**Topics of Public Interest:**

Agricultural law, the amended.. 161
Consumption catechism for children ..................... 158
Consumptives, old ferryboats used in treating .............. 41
Draper, Andrew S., address before New York State Historical Association ............ 159
Education in England ......... 392
Medical education in United States, Great Britian and Germany .................... 33
Medical examiners, new state board of .................... 97
Medical profession in legislative bodies ........................ 709
Money, no disease on .......... 333
Navy medical corps (U.S.) gets a stimulus .................. 404
Prizes on tuberculosis ......... 531
Secretary of a county medical society ...................... 103
Substitution, to prevent ....... 102
Tuberculosis, Buffalo to fight... 707
Typhoid from oysters ......... 223

Tramp life studied by a student.... 677
Tubes and ovaries, treatment of ... 600
Tuberculosis, an infectious disease.. 668
cows and tuberculosis in children ......... 709
feeding in ........... 440
meeting in reference to ................ 417
warfare against ..... 343

UTERINE hemorrhage, treatment of ......................... 497
Uterus, curetting the ............. 654
retrodeviations of ......... 584
treatment of prolapse of... 565

VAGINAL Cesarean section ..... 644
Varicose veins ............... 621
Van Peyma, P. W., neglect of scientific obstetrics ................. 125
Venereal disease, ethical side of professional secrecy in .............. 154
Voice, misuse of and cure ......... 288

WATER, pure at Niagara Falls .. 609
Wells, William H., obstetric and version ..................... 274
West, James N., treatment of prolapse of uterus .................. 565
Wiley, H. W., expulsion of drugs by kidneys ......................... 479
Williams, Herbert U., acute pancreatitis ............................ 689
Winter sports and accidents ....... 478
"Woman in medicine"—Maud J. Frye ......................... 233
Women, laurels for medical ....... 713
Osler on medical ......... 156

